# Supplementary material for: Comparison of [18F]FDG PET/CT and MRI for Treatment Response Assessment in Multiple Myeloma: A Meta-Analysis
Source: Diagnostics (Basel). 2021 Apr 15;11(4):706. doi: 10.3390/diagnostics11040706 (PMC8071116; doi:10.3390/diagnostics11040706)
Supplement: Supplementary file 1 [file diagnostics-11-00706-s001.zip › diagnostics-1160739-supplementary.pdf]

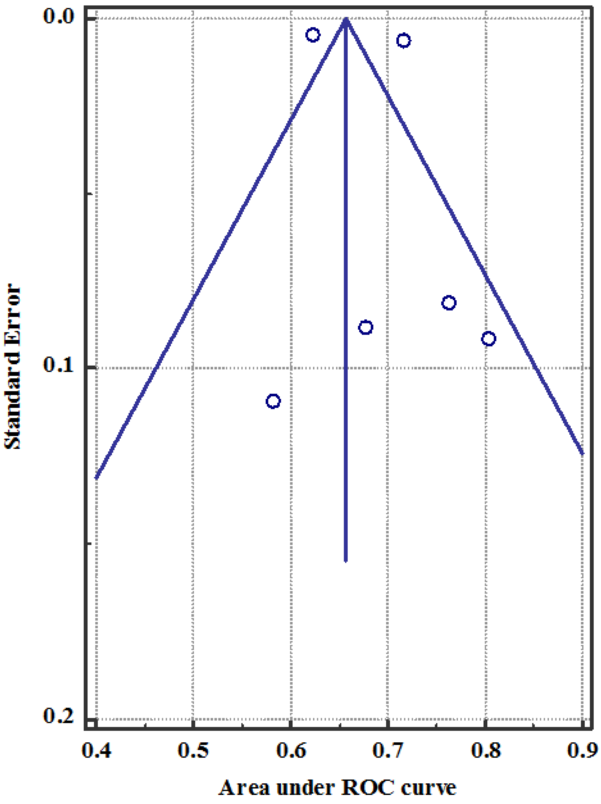

Funnel plot of AUC value in FDG PET/CT

Publication bias

Egger's test	
Intercept	1.3412
95% CI	-7.1967 to 9.8790
Significance level	P = 0.6852
Begg's test	
Kendall's Tau	-0.06667
Significance level	P = 0.8510

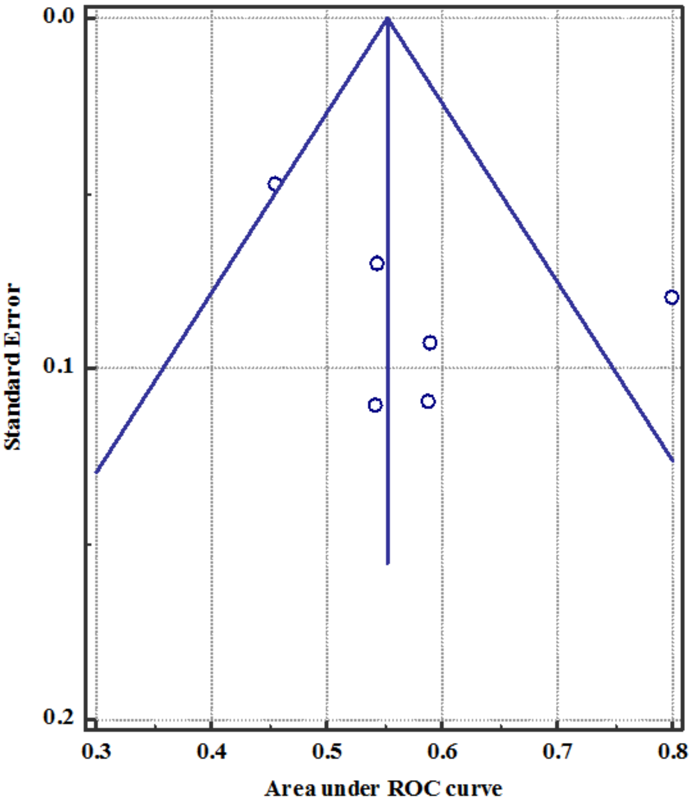

Funnel plot of AUC value in MRI

Publication bias

Egger's test	
Intercept	2.7068
95% CI	-3.0955 to 8.5091
Significance level	P = 0.2649
Begg's test	
Kendall's Tau	0.2000
